# Home care aides’ observations and machine learning algorithms for the prediction of visits to emergency departments by older community-dwelling individuals receiving home care assistance: A proof of concept study

**DOI:** 10.1371/journal.pone.0220002

**Published:** 2019-08-13

**Authors:** Jacques-Henri Veyron, Patrick Friocourt, Olivier Jeanjean, Laurence Luquel, Nicolas Bonifas, Fabrice Denis, Joël Belmin

**Affiliations:** 1 Agence Nationale d’Appui à la performance (ANAP), Paris, France; 2 Service de médecine interne gériatrique, Hôpital Simone Veil, Blois, France; 3 Pôle de gériatrie, SSR, Soins Palliatifs, Groupe hospitalier Nord-Essonne, Longjumeau, France; 4 Hôpital privé gériatrique Les Magnolias, Ballainvilliers, France; 5 Ecole Polytechnique, Palaiseau, France; 6 Institut Inter-Regional de Cancérologie Jean Bernard, Le Mans, France; 7 Hôpital Charles Foix, Assistance Publique-Hôpitaux de Paris, Ivry-sur-Seine, France; 8 Sorbonne Université, Paris, France; Medical University Graz, AUSTRIA

## Abstract

**Background:**

Older individuals receiving home assistance are at high risk for emergency visits and unplanned hospitalization. Anticipating their health difficulties could prevent these events. This study investigated the effectiveness of an at-home monitoring method using social workers’ observations to predict risk for 7- and 14-day emergency department (ED) visits.

**Methods:**

This was a prospective cohort study of persons ≥75 years, living at home and receiving assistance from home care aides (HCA) at 6 French facilities. After each home visit, HCAs reported on participants’ functional status using a smartphone application that recorded 27 functional items about each participant (e.g., ability to stand, move, eat, mood, loneliness). We recorded ED visits. Finally, we used machine learning techniques (i.e., leveraging random forest predictors) to develop a 7- and 14-day predictive algorithm for the risk of ED visit.

**Results:**

The study included 301 participants, and the HCA made 9,987 observations. Over the mean 10-month follow-up, 97 participants (32%) had at least one ED visit. Modeling techniques identified 9 contributory factors from the longitudinal records of the HCA and developed a predictive algorithm for the risk of ED visit. The predictive performance (i.e., the area under the ROC curve) was 0.70 at 7 days and 0.67 at 14 days.

**Interpretation:**

For frail elders receiving in-home care, information on functional status collected by HCA helps predict the risk of ED visits 7 to 14 days in advance. A survey system for real-time identification of risks could be developed using this exploratory work.

## Introduction

Visits to the emergency department (ED) and the consequent hospital admissions among elderly adults are an important public health issue [1,2]. The number of ED visits has sharply increased in recent years [3]. In the US, almost one of every three US Emergency Medical Services (EMS) emergency responses involves an older adult [4], and this number is expected to continue to grow as the baby-boomer generation ages.

Prevention of unplanned hospital admission is a major focus for the treatment of the elderly. Since a large proportion of ED visits are avoidable [5–7], strategies to identify high-risk patients and enable them to be treated in outpatient care settings might help to improve the appropriate use of EDs and to control health expenditures related to EDs [8]. Even when hospitalization is required, a planned admission in an appropriate ward is a better option than is being hospitalized following an ED visit [9], as long as the patient is not in a life-threatening situation. Accordingly, developing methods of predicting ED visits and identifying at-risk persons is a very promising research approach [10,11]. Particularly, patient symptom monitoring by e-health tools might improve survival and reduce the need for ED visits [12,13].

Emergencies among the elderly are mainly the result of complex situations that often comprise an acute event—the crisis—and a chronic vulnerable state that forms through the accumulation of chronic conditions frequently encountered in this population, such as medical diseases and social, psychological, or socio-economic frailty [1]. Several studies have identified major risk factors for ED visits [7,14–16]. Based on the results of these studies, prediction tools for identifying risks for ED visits or hospitalizations have been developed. These tools include the Community Assessment Risk Screen (CARS), a simple tool to identify elderly persons at risk for ED visits or hospitalization [17]; the Elder Risk Assessment Index [14]; and the Adjusted Clinical Groups (ACG) [15]. All three tools are based on individual characteristics recorded at a single point in time, and have been found to be effective in predicting this risk in the subsequent 12–24 months (with a predictive performance of around 0.70, as assessed by the area under the curve of the receiver operating characteristic [ROC] model). However, to our knowledge, researchers have not developed a tool capable of predicting the risk of ED visits over a shorter period.

Nurse aides can accurately use well-calibrated instruments to assess nursing home residents' functional health [18]. In the present study, we were interested in whether observations recorded by home care aides (HCAs) using smartphone technology were capable of predicting ED visits for frail adults aged 75 or older who were receiving in-home help. We hypothesized that the longitudinal monitoring of simple items related to everyday life and recorded by HCAs during their visits could detect changes in the functional status of older adults and could contribute to identifying items related to acute or subacute health problems, and thus contribute to predicting ED visits over a short period.

## Materials and methods

### Design and participants

In this prospective observational cohort study, participants were recruited from among adults aged 75 and over who were living at home and receiving the assistance from an HCA at one of the six home care services located in three French departments (Essonne, Val de Marne, Loir-et-Cher). These HCAs were not healthcare professionals and typically provided assistance with non-medical tasks (e.g., help with meals, assisting with personal care, housekeeping, running errands). Dependency levels of the persons were established according to the French national instrument, which stratifies dependency level from *groupe iso-ressources* (GIR) 1 (very severe dependency) to GIR 6 (no dependency) [19]. Persons with severe dependency, or those with a GIR 1 or GIR 2 dependency level, were excluded from the study since most of them were receiving daily nursing care involving surveillance and alerts to medical staff. For people with low dependency (GIR 3 to 5), daily nursing care is much less frequent, and systematic alerts to medical staff are difficult to follow. All eligible persons were invited to participate and were included if they provided consent. We identified 350 eligible persons at the participating facilities, and 301 of them (86%) consented to participate. The recruitment process occurred between June 6, 2016 and July 27, 2017, and the home visit recordings by the HCAs started on September 1, 2016 and ended on January 19, 2018.

### Ethics

The research protocol was approved by the official French independent ethics committee for biomedical research, the Comité de Protection des Personne Ile-de-France VI. Prior to the start of the study, participants and HCAs were informed about the nature and purpose of the study, and all provided their written consent.

### HCA recruitment and demographics

A total of 142 HCAs were employed by six different home care services, of which 136 (95.7%) were enrolled in the study by home care services managers. Table 1 shows the demographics of HCAs (the specific home care services were anonymized using the notations N°1 to 6).

**Table 1:** Demographics of home care aides.

	Variables	Values	
**Home Care Assistant**	Age (years, mean, SD)	34	9.5
	Home Care Services (n, %)		
	N° 1	22	16.2
	N°2	2	1.5
	N°3	18	13.2
	N°4	29	21.3
	N°5	40	29.4
	N°6	25	18.4
	All	136	100
		Gender (n, %)		
		Female	135	99.2
		Male	1	0.8

### Data collection

Immediately after participants consented to participate, we obtained information on their demographics, home characteristics, medical conditions, and medical and paramedical support from the home help facility. All this information was recorded by a coordinator on a questionnaire comprising 23 items. Table 2 shows 15 items with a completeness rate of 60% or more; the supplemental material lists the eight remaining variables (S1 Table).

**Table 2:** Baseline characteristics of participants and home care organizations (as recorded by home care service coordinators) and their completeness rates.

	Variables	Completeness	Values	
		%	n	%
**Participants**	Age distribution (years)	100		
	75–79		27	9.0
	80–84		41	13.6
	85–89		92	30.6
	≥ 90			
		14	46.8
	Gender	100		
	Female		226	75.1
	Male		75	24.9
	Dependency level	100		
	Moderate (GIR 3 or 4)		145	48.2
	Mild (GIR 5 or 6)		156	51.8
	Living alone	96.3		
	Yes		212	70.4
	No		78	25.9
	Has a family caregiver	94.8		
	Yes		212	70.4
	No		78	25.9
	Beneficiary of care for ALD chronic diseases	74.1		
	Yes		94	31.2
	No		129	42.9
**Home characteristics**	Home is accessible to the person	95.3		
	Yes		274	91.0
	No		13	4.3
	Bathroom is adapted to the person	95.0		
	Yes		261	86.7
	No		25	8.3
	Home spaces are cluttered	91.7		
	Yes		37	12.3
	No		239	79.4
**Care organization**	Physician home visit frequency >1 per 3 months	64.5		
	Yes			
	No		79	26.2
			115	38.2
	Paramedical home health care intervention	73.8		
	Yes			
	No		104	34.6
			118	39.2
	Home visits per week by the home care aides	87.0		
	1–2			
	3–4		155	51.5
	>4		42	14.0
			65	21.6
	Duration of home care visits per week	72.7		
	Less than 5 hours		161	53.5
	5–10 hours		64	21.3
	>11 hours		6	2.0
	Type of aid provided (multiple choices possible)	77.0		
	Housekeeping			
	Meals		202	67.1
	Errands		21	7.0
	Personal care		54	17.9
			73	24.1
	Weekend visits	73.8		
	Yes		33	11.0
	No		186	61.8

Note: ALD: list of chronic diseases defined by the national health insurance service in France; GP: general practitioner; GIR: groupe iso-ressources, an indicator of the level of dependency)

We asked the HCAs to work as they usually do except for the data collection. At the end of each home visit, the HCAs recorded information about the participants via a smartphone application developed by one of the authors. First, the HCA took a photo of the personal QR code assigned to each participant, which we had placed in his/her home. This recorded the participant’s name, date, and time of the visit. Second, the HCA answered 27 simple questions related to the patient’s functional status, behavior, or support for help (Table 3). For each question, the possible answers were *yes/no/do not know*. We explained the purpose of the study to the HCAs, as well as providing training and a user manual on how to use the application and the exact meaning of each term used in the application. The application ensured real-time data transmission to the investigators without further dissemination, which we explained to the HCAs. The average observation time for participants was 10.7 months (SD = 3.5 months).

**Table 3:** List of 27 items recorded by the home care aides at each home visit and their completeness rates.

Items related to	Items	Completeness Rate (%)
Activities of daily living	The person has groomed him/herself	93
	The person gets out of bed	64
	**The person is able to move in his/her home**	96
	The person has moved out of the home	83
	**The person has prepared his/her meal**	94
	The person has eaten	66
Possible medical symptoms	The person seems tired	88
	**The person seems feverish**	94
	**The person is painful**	82
	**The person has trouble breathing**	95
	The person has swollen legs	93
	**The person has fallen**	69
	The person seems better than at the last visit	99
Behavioral troubles	The person places objects in inappropriate places	66
	The person is aggressive	95
	The person does not recognize me	94
	The person has forgotten when I came	94
	The person has refused help for grooming	90
	The person has refused interventions for help	91
	The person communicates inconsistently	94
Communication—entourage	**The person communicates little**	95
	**The person seems sad**	88
	The person seems indifferent	95
	**The person has no visit from, or contact with his/her social support**	95
	The family caregiver seems sad	100
	The family caregiver seems exhausted	100
	The family caregiver is gone or absent for several days	86

Note. For each item, the possible answers were: *yes*, *no*, *do not know*. The nine items figured in bold were found to contribute to the 7-day and 14-day models predicting the visit to emergency departments.

With the help of EDs around the facilities, we identified participants’ ED visits using systematic reviews of their registers. For each ED visit, an investigator recorded the date, the reasons for the visit, and the visit outcome (i.e., hospital admission or not).

### Predictive modeling for ED visits

We randomly selected participants to form the training sample used to build predictive models and then assessed the relevance of the models from the remaining participants (the test sample) with the events that had not been used to build the model. We excluded poorly filled variables (i.e., completeness below 60%) from the modeling.

Using machine learning techniques (random forests), we developed several predictive models using baseline data and the home visits variables to predict ED visit with a 7 or 14-day horizon. In order to respect the proportions between ED visits and no ED visits, the learning sample was composed of 1000 negatives / 20 positives and the test sample of 500 negatives / 10 positives.

Note that several machine learning techniques were actually attempted, including linear and logistic regressions, as well as random forests. However, we obtained the best results, particularly in terms of generalization, with the random forests. It is also noteworthy that we engineered the features passed on to the machine learning model. That is, we built a so-called temporal variance matrix that aggregates all the questions into one value for each week and learned from the evolution of that value from week to week. The best results were obtained when making predictions using those features.

We evaluated the performance of the models according to the Gini index (the area under the curve [AUC]) as well as the local area under the curve (for the 1% of individuals predicted to have the highest ED visit risk).

## Results

### Participants

Table 2 details the characteristics of the participants and the home care organization. The participants’ mean age was 88 years (SD = 5.8 years), and the sample included 226 women (75.1%). During the study period, 32 participants (11%) dropped out from the study before the end of the follow-up, and among them, 19 had an ED visit before dropout. The reasons for dropouts were death (n = 14), nursing home admission (n = 5), and other reasons such as stopping home help service (n = 13).

### Feasibility and completeness of records

There was heterogeneity in the completeness of the baseline data recorded by the home care service coordinators, ranging from 20% to 100%. Table 2 lists the variables related to dependency and the social factors with high completeness rates. By contrast, the variables related to medical status, like the number of prescribed drugs, history of previous hospital admission or emergencies visits, as well as the occurrence of actual paramedic interventions had a low completeness rate. There were eight variables with a completeness rate below 60% (listed in supplements), which we excluded from the modeling.

Using smartphones, 136 HCAs recorded data from 9987 home visits, which corresponds to 31% of the expected home visits during the follow-up period. The mean completeness rate of variables was 85%, and no variable had a completeness rate of < 60% (Table 3). Thus, all the variables were included in the models.

### ED Visits and hospital admissions

During the follow-up, 97 participants (32.5%) visited EDs, and among them, 35 went two or more times (up to 5 visits); thus, overall, 152 ED visits were recorded (Table 4). Hospital admission following an ED visit occurred for 60 participants (20% of the overall cohort and 61% of those who visited an ED). During the same period, 19 participants were hospitalized by a direct admission without any ED visits.

**Table 4:** Emergency department (ED) visits and hospital admission of the 301 participants during the follow-up.

	Participants	Number of Events
	(N = 301)	
ED visit	97 (32.5%)	152
No hospital admission after ED visit	48 (16%)	68
Hospital admission after ED visit	50 (17%)	86
Discharge to home after hospital stay	31 (10%)	64
Transfer to rehabilitation settings or another hospital ward (surgery, …)	19 (6%)	22
Direct hospital admission (no ED visit)	14 (5%)	19
Discharge to home	3 (1%)	11
Transfer to rehabilitation settings or another hospital ward (surgery …)	7 (2%)	8
Any hospital admission	64 (20%)	105
Discharge to home	34 (11%)	75
Transfer to rehabilitation settings or another hospital ward (surgery …)	26 (9%)	30

Note. ED, emergency department.

### Construction and evaluation of the predictive models

**Fig 1:**
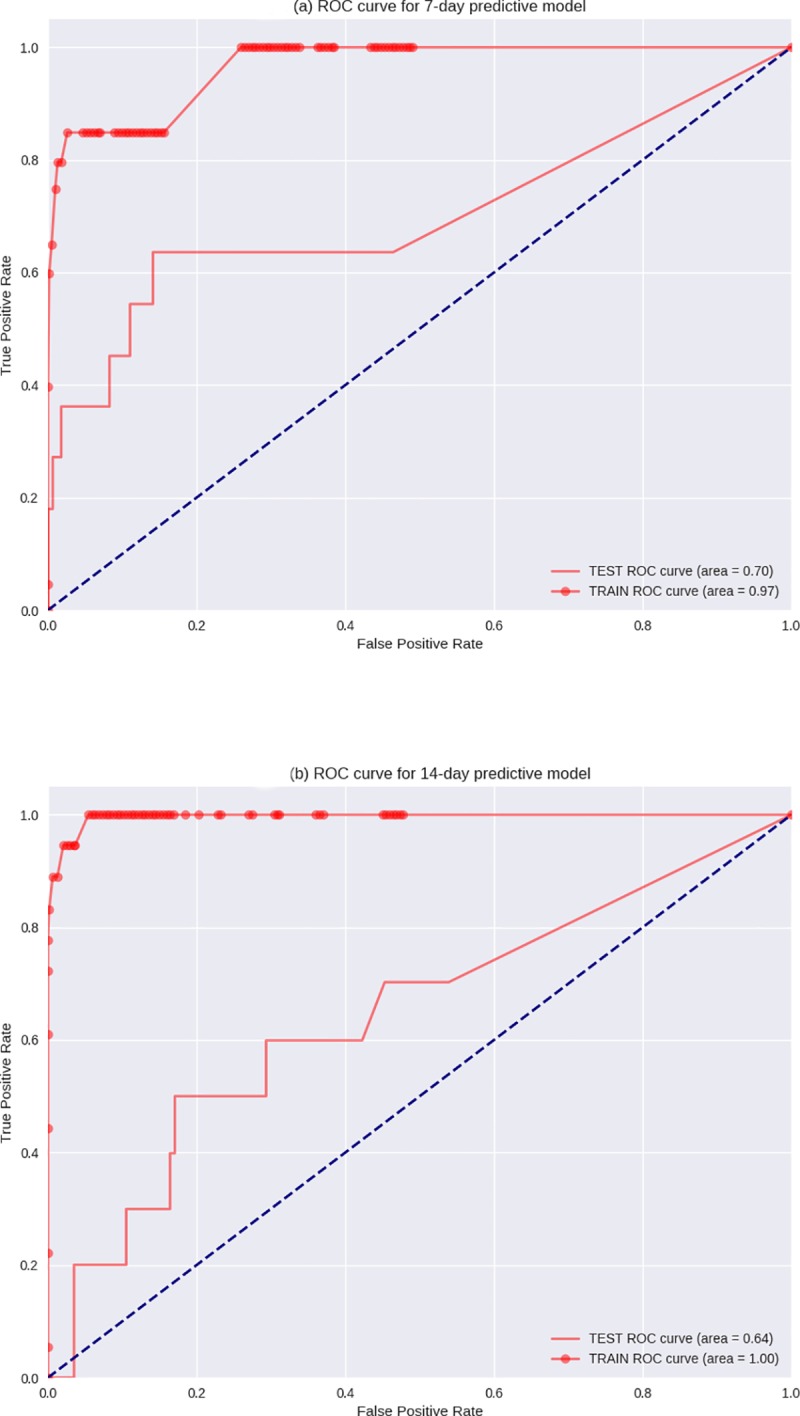
(a) ROC curve for 7-day predictive model and (b) ROC curve for 14-day predictive model.

First, we compared participants’ characteristics according to the presence/absence of ED visits during follow-up (Table 5). Then, using all the variables selected based on completeness, we developed several predictive models and assessed their performance in predicting 7-day and 14-day visits to ED. We obtained the best 7-day predictive model by using the data from nine variables recorded by the HCA observations (Table 3), whereas the data from baseline variables did not improve the accuracy of the models. The global performance assessed by the area under the curve of the ROC curve was 0.70 for the 7-day predictive model and 0.63 for the 14-day predictive model. The slope of the curve between the origin and 3% (positive likelihood ratio) for the 7-day model was 16.4 (Fig 1A and 1B). For the 7-day model, the sensitivity and specificity were 36% and 98%, respectively. The positive predictive and negative predictive values were 21% and 99%, respectively.

**Table 5:** Comparisons of participants’ baseline characteristics according ED visits or no ED visit during the follow-up.

	ED visit	No ED visits	P value
	(n = 97)	(n = 204)	
Age (years) mean (SD)	88.1 (5.70)	88.5 (5.84)	0.336
Female (n, %)	76, 78.4	150, 73.5	0.366
Dependency level (n, %)			0.299
Moderate (GIR 3 or 4)	43, 44.4	102, 50.0	
Mild (GIR 5 or 6)	54, 55.6	102, 50.0	
Living alone (n, %)	73, 75.3	132, 64.7	0.431
Has a family caregiver (n, %)	66, 68.0	146, 71.6	0.923
Beneficiary of care for ALD chronic disease(s) (n, %)	28, 28.9	43, 44.3	0.575
Home is accessible to the person (n, %)	86, 88,7	188, 92.2	0.527
Home is adapted to the person (n, %)	85, 87.6	176, 86.2	0.087
Home spaces are cluttered (n, %)	15, 15.5	22, 10.8	0.136
GP home visit frequency > 1 per 3 months (n, %)	34, 35.1	45, 22.1	0.053
Paramedical home health care intervention (n, %)	32, 33.0	72, 35.3	0.619
Home visits by the home care aides (n per week), mean (SD)	2.61 (2.02)	2.87 (1.97)	0.216
Duration on home care visits (hours per week), mean, SD)	3.27 (2.50)	4.10 (2.88)	0.432
Weekend visits (n, %)	8, 8.2	25, 12.3	0.390

Note. SD, standard deviation; ED, emergency department.

Among the nine variables having a significant contribution to the realized model (Table 3), two were related to activities of daily living (i.e., the person is able to move in his/her home, has prepared his/her meal), four to possible medical symptoms (i.e., the person seems feverish, is painful, has trouble breathing, has fallen), and the remaining three to communication and social support (i.e., the person communicates little, seems sad, has no visit of, or contact with family members).

## Discussion

In this proof of concept study with frail elderly persons who were receiving home help (social assistance), we found that it is feasible to organize the longitudinal recording of patients’ functional status by their HCAs over a period of several months, by using a simple smartphone application at each home visit. We also found that these records can predict the risk of ED visit within the following 7 and 14 days. Our findings open the way to develop an innovative approach for the implementation of new systems for real-time monitoring of the risk for ED visits and to expand anticipatory interventions that decrease the risk for unplanned hospital admission in this high-risk population.

We conducted our study with elderly participants (i.e., ≥75) who were experiencing mild to moderate dependency and receiving home care. Their risk for ED visits or hospital admission was high, as indicated by the fact that 33% visited an ED, and 20% were hospitalized during this 10-month survey. A large study by Jones et al. [20] with a cohort of 32,253 elderly persons receiving home care in Ontario also observed a high incidence rate of ED visits and hospitalizations, even if those rates were lower than seen in our sample. We took advantage of the help delivered by home care services by asking HCA to record simple items related to the functional status of the participants at each home visit. The high rate of participation of the aides is a strength of our study since HCA are not health care professionals and have typically not been involved in monitoring symptoms and participating in research [21]. The successful participation in this study is likely due to the ease of completion, with simple items and the use of a friendly app installed on a smartphone. For those elderly persons using in-home care, the involvement and empowerment of HCAs in health monitoring is a promising way to improve the management of chronic disease and medical events without increasing health expenditures cost. To our knowledge, researchers have not reported such an approach for home care, and our study shows that HCA involvement is feasible even over a long period of time.

In our study, we obtained longitudinal data on the functional status of the participants, and in line with our hypothesis, found that changes in several variables were predictive of a 7- or 14-day ED visit. Interestingly, the predictive variables comprise items related to possible medical symptoms (e.g., having trouble breathing), to functional status (e.g., changes in the ability to move in the home) as well as items related to mood problems or loneliness. All these items might reflect the initiation of a health event and/or a worsening of the health status, which might expose the participant to a crisis and a visit to ED. Jones et al. [20] observed that, among people receiving home care, the likelihood of visiting the ED was greater on the day of the home visit compared to the days without a visit. This was probably related to the identification of medical problems by HCAs when they visit participants in their care, which can lead to the alerting of patients’ family caregivers or physicians, and subsequently to ED visits. However, this association was observed only with nursing visits but not with HCA visits, probably because nurses are more capable of recognizing medical problems and alerting others when required.

The AUC (0.70) in our study was comparable to those found in other state-of-the-art studies using machine learning techniques to predict future behavior. Recent examples include the prediction of sepsis with an AUC of 0.67 [22] and the prediction of delirium with an AUC of 0.68 [23]

More importantly, the slope of the ROC at origin for the 7-day model was 16.4, and had a positive likelihood ratio of 3%. This shows that we can very accurately predict a certain fraction of ED visits. Certain causes of ED visits (e.g., acute decompensation, accidents) could not be foreseen by any means one week in advance. Thus, the sensitivity reflects the fact that we predict a complex response (all causes of ED visits within seven days) from very little data (27 simple observations, once per week). We further believe that a sensitivity of 36% is very significant already. In addition, further studies could establish a threshold value, which results from the status evaluation by social workers. Medical staff (e.g., nurses) should visit the respective person on the very same day and after further investigation should have to decide whether a family doctor must be summoned, whether an immediate clinic visit is necessary, or whether an appointment (e.g., in specialty clinics) would have to be made on the following day or in the next few days.

The study has a number of limitations. First, we had little medical information from participants, although several medical factors were found to predict emergency room visits over a 12-month period.[16,24–26]. In our study, we attempted to record information about participants’ medical status on our baseline questionnaire by the home care services coordinators, but we obtained a low completeness rate. This rate might be because these professionals had a low degree of knowledge regarding the medical status of the participants. Despite this limitation, it is impressive that the longitudinal records on functional status were a significant predictor of ED visits in the short term, even without precise information about their chronic diseases, drug utilization, and previous hospitalizations or ED visits. To the extent that the nature of the information recorded by home support workers is very different from that contained in conventional medical information, we believe that combining the two types of information could improve the predictive model. Second, the sample size of our study is small to conduct powerful machine learning studies. However, the promising results of this proof-of-concept study encourage us to develop this approach in larger populations, which will lead not only to validate the approach, but also to improve the predictive model.

These findings highlight a novel approach to improving the predictive performance of the models and recognizing more efficient tools for the short-term prediction of ED visits—namely, by including both baseline records about chronic diseases and longitudinal records about symptoms and functional status.

In summary, we conclude that HCA observations can successfully contribute to the short-term prediction of ED visits by elderly persons receiving home care and that this approach together with long-term prediction models might constitute a novel method of predicting ED visits. This method may promote real-time interventions capable of reducing avoidable ED utilization in this high-risk population.

## Supporting information

S1 Table List of the eight baseline items with a completeness rate below 60%. These variables were not considered in the models.
(DOCX)

S2 Table Data for 7-days ROC and 14-days ROC. (XLSX)

S3 Table Strobe check-list. (DOCX)
