# Supplementary material for: Home care aides’ observations and machine learning algorithms for the prediction of visits to emergency departments by older community-dwelling individuals receiving home care assistance: A proof of concept study
Source: PLoS One. 2019 Aug 13;14(8):e0220002. doi: 10.1371/journal.pone.0220002 (PMC6692042; doi:10.1371/journal.pone.0220002)
Supplement: S1 Table — These variables were not considered in the models. (DOCX) [file pone.0220002.s001.docx]

| **Items** | **Completeness rate (%)** |
| --- | --- |
| Frequency of general practitioner visit at home (n per trimester) | 58% |
| Number of prescribed drugs (n) | 20% |
| Involuntary weight loss (yes/no) | 59% |
| Involuntary rapid weight loss (yes/no) | 56% |
| Number of hospital admissions in the past 6 months (n) | 20% |
| Number of ED in the past 6 months (n) | 23% |
| Health care professionals intervening at home (n and type) | 48% |
| Health care professionals’ home visits (n per type) | 40% |
